# Diagnosis and Differential Diagnosis of Neurological Adverse Events during Immune Checkpoint Inhibitor Therapy

**DOI:** 10.1155/2020/8865054

**Published:** 2020-12-07

**Authors:** Nora Möhn, Susann Mahjoub, Ralf Gutzmer, Imke Satzger, Gernot Beutel, Philipp Ivanyi, Heiko Golpon, Mike P. Wattjes, Martin Stangel, Thomas Skripuletz

**Affiliations:** ^1^Department of Neurology, Hannover Medical School, Hannover, Germany; ^2^Immune Cooperative Oncology Group (ICOG), Comprehensive Cancer Center Niedersachsen (CCCN), Hannover, Germany; ^3^Skin Cancer Center Hannover, Department of Dermatology and Allergy, Hannover Medical School, Hannover, Germany; ^4^Department of Hematology, Hemostasis, Oncology and Stem Cell Transplantation, Hannover Medical School, Hannover, Germany; ^5^Department of Pneumology, Hannover Medical School, Hannover, Germany; ^6^Department of Diagnostic and Interventional Neuroradiology, Hannover Medical School, Hannover, Germany

## Abstract

Therapy with immune checkpoint inhibitors (ICIs) has improved overall survival and cancer-related morbidity of cancer treatment even in cancer entities with poor prognosis. Since the approval of the first ICI, ipilimumab, for treatment of advanced melanoma by the Food and Drug Administration (FDA) in 2011, the spectrum of indications and approved ICIs has grown, rapidly. Up to now, seven different ICIs for more than 20 indications are available. However, their mechanisms of action can lead to immune-related adverse events (irAEs). In particular, neurological irAEs are clinically relevant. Although they are rare, an early and accurate diagnosis is challenging and neurological disease course and sequelae are potentially fatal. Between 08/2017 and 03/2020, 31 patients received ICI treatment at Hannover Medical School and presented with neurological adverse events (N-irAEs). Treated malignancies were metastatic melanoma, bronchial carcinoma, and urothelial cell carcinoma. All patients received comprehensive neurological diagnostics including clinical examination and magnetic resonance imaging (MRI). Cerebrospinal fluid (CSF) analysis was obtained in 21 patients and electroneurography was performed in 22 patients. Although N-irAEs were suspected in all 31 patients, 11 patients had other conditions leading to neurological symptoms including tumor metastases in seven patients and hemorrhagic or ischemic stroke in four patients. In the following, these patients are referred to as the differential diagnosis (DD) group. Patients with N-irAEs suffered from immune mediated neuropathy (9/20), myositis and/or myasthenic syndrome (6/20), or encephalitis/cerebellitis (5/20). Except for cell count, CSF results did not differ between the N-irAEs and the DD group. Symptoms related to N-irAEs are rather unspecific potentially mimicking other tumor-related symptoms such as metastases. Patients with malignancy are predominantly not treated by neurologists. Because of the complexity of neurological symptoms, detailed neurological investigations in specialized institutions are necessary in patients with new neurological symptoms and need to be critically discussed with treating oncologists.

## 1. Background

Utilizing the body's own immune system rather than attacking cancer cells with conventional chemotherapy, radiotherapy, or surgery has been a preferable treatment strategy for many years. Recently, immunotherapy has demonstrated a new path to improve clinical outcome of cancer patients [1]. Cancer cells are able to evade anti-tumor response by overexpressing immunosuppressive molecules such as cytotoxic T-lymphocyte associated antigen 4 (CTLA-4), programmed cell death protein (PD-1), and its ligand PD-L1 [2]. Under normal conditions, immune checkpoints regulate immune responses, maintain self-tolerance, and prevent autoimmunity through inhibiting T-cell activation [3]. Blocking these immune checkpoints by immune checkpoint inhibitors (ICI) increases T-cell immune response against tumor cells. Up to now, eight ICIs have been approved for the treatment of different cancers such as melanoma, non-melanoma skin cancer (Merkel cell carcinoma and cutaneous squamous cell carcinoma), bladder, bronchial, head and neck, and renal cell carcinoma, as well as breast cancer and Hodgkin diseases [4–8]. However, ICIs may disrupt the balance between activation and suppression of the immune system and favor the development of autoimmune diseases [9–12]. Side effects termed as immune-related adverse events (irAEs) may affect every organ. Most commonly, the dermatologic, gastrointestinal, pulmonary, hepatic, and endocrine systems, as well as constitutional symptoms, are involved [13]. Neurological adverse events due to ICI therapy (N-irAEs) are relatively rare but can be potentially fatal leading to considerable sequelae despite adequate therapy [14]. Underreporting of N-irAEs related to ICI therapy can be anticipated because symptoms can be unspecific and difficult to interpret [15]. Additionally, autoimmune side effects of ICI therapy may occur even months after completion or termination of immunotherapy which makes the correct diagnosis even more difficult [16]. They can involve every part of the central and peripheral nervous system [17, 18], whereby neuromuscular complications are the most common [17, 19, 20]. Interestingly, patients with neuromuscular manifestations often do not present with specific symptoms and laboratory signs mimicking other diseases such as myasthenia gravis, Guillain–Barré syndrome, and myositis [21]. Central nervous system (CNS) manifestations of N-irAEs are mostly described in small case studies. However, one conclusive fact applies to all N-irAEs: the underlying mechanisms and their pathogenesis have not been fully elucidated yet. Guidelines for the management of N-irAEs rely mostly on expert opinions and refer to the knowledge of other autoimmune diseases [14]. Besides, the fact that diagnosis and management of N-irAEs can be challenging, a major task is to distinguish between an immune-related neurological condition and other differential diagnoses such as symptomatic metastases, paraneoplastic effects, or even neurovascular diseases. Other potential causes need to be excluded before patient's symptoms can be considered as N-irAEs. However, we have to keep in mind that autoimmune side effects are usually overlooked rather than over-diagnosed. The aim of this study was to investigate a cohort of 31 patients with different underlying cancers who were treated with ICIs and presented with new neurological symptoms. Although N-irAEs were suspected as the initial reason for patient's symptoms, a high number suffered from direct cancer associated disorders underscoring the importance of differential diagnoses.

## 2. Methods

### 2.1. Study Design and Patient Selection

Between 08/2017 and 03/2020, 31 patients with neurological symptoms during or after ICI therapy treated at Hannover Medical School have been identified by their attending physicians. Due to N-irAEs, patients were treated at the Department of Neurology or the attending department received neurological support. For the purpose of this study, patients were included retrospectively. Eight patients included in the present study were previously described in detail [21].

### 2.2. Participants

Demographic data collected included age, sex, underlying disease, duration of ICI therapy, number of ICI cycles, symptoms of neurological manifestation, type of potential immunosuppressive therapy, and date of final diagnosis. Data were obtained from electronic patients' records. Diagnostic data included brain and spinal cord magnetic resonance imaging (MRI) findings, electrophysiological diagnostics (EEG, ENG, EMG), CSF parameters (cell count, protein levels, lactate, intrathecal immunoglobulin synthesis, oligoclonal bands (OCBs)), and serum analysis for certain antibodies (myositis-, antineuronal-, autoimmune encephalitis- and anti-AChR-antibodies). All patients gave written informed consent for publication (approved by the ethics committee of Hannover Medical School, vote number 2413–2014). Analyses were done in concordance with local ethic committee recommendation respecting the Declaration of Helsinki in its latest version.

### 2.3. Diagnostic Procedures

CSF and serum were analyzed by routine methods which have been described before [22]. Immediately after CSF withdrawal by lumbar puncture, CSF cell count, total CSF protein, and CSF lactate were analyzed. Cell count was manually determined with a Fuchs–Rosenthal counting chamber. For cytology, cells were stained with the Pappenheim method, a combination of May–Grünwald (Merck, Darmstadt, Germany) and Giemsa staining (Sigma-Aldrich, St. Louis, USA). In individual cases, melanin staining of CSF cells was performed in order to confirm the diagnosis of meningeosis neoplastica. For further analyses, the residual CSF was centrifuged (145 g for 15 min) and the supernatant frozen at −70°C. CSF oligoclonal bands were determined by isoelectric focusing in polyacrylamide gels with consecutive silver staining. Five patterns of oligoclonal bands were distinguished following the recommendations of the first European consensus on CSF analysis in multiple sclerosis [23].

Commercially available cerebellum primate slides (INOVA Diagnostics) and immunoblots with recombinant antigens (PNS-Blot, Ravo Diagnostika) were utilized for detection of antineuronal antibodies (anti-Yo, -Hu, -Ri, -CV2, -Ma1/2, -SOX1, -Tr, -CARP, -ITPR1, -GAD, ZIC4, and -amphiphysin antibodies) via indirect immunohistochemistry. Myositis antibodies were detected via immunoblot method using test strips coated with Mi-2*α*, Mi2*β*, TIF1*γ*, MDA5, NXP2, SAE1, Ku, PM-Scl100, PM-Scl75, Jo-1, SRP, PL-12, EJ, OJ, and Ro-52 antigens (EUROLINE autoimmune inflammatory myopathies 16 Ag, Euroimmun AG). For analysis of autoimmune encephalitis antibodies (anti-NMDA-, -CASPR2-, -AMPA1/2-, -LGI1-, -DPPX-, and -anti-GABA B receptor-antibodies), transfected cells on BIOCHIPS (Euroimmun AG, Lübeck, Germany) were incubated with patient sera. Quantification was obtained via the specific fluorescence intensities using an indirect immunofluorescence test [24]. Serum-anti-acetylcholine receptor (AChR) antibodies were quantified via radioimmunoassay and ganglioside antibodies via enzyme-immunoassay.

Brain and spinal cord MRI were performed by using 1.5 Tesla machines and included T1-weighted, T2-weighted, fluid-attenuation inversion recovery (FLAIR), and diffusion-weighted (DWI) sequences. In certain cases, susceptibility-weighted images (SWI) were added. Spinal imaging included sagittal and axial T1-weighted and T2-weighted sequences, respectively.

Electrophysiological diagnostics were performed with superficial stimulators and recording electrodes as conventional standard routine procedures according to the recommendations of the International Federation of Clinical Neurophysiology [25].

### 2.4. Statistics

Statistical analysis was performed using GraphPad Prism Software version 8.0 (GraphPad Software, San Diego, USA). Appropriate statistics were administered for subgroup comparison. *p* < 0.05 was considered statistically significant.

## 3. Results

### 3.1. Patient's Characteristics

The median age of our patients was 67 years (range: 23–89) and 20/31 patients (65%) were male. Underlying malignancies were melanoma (18), bronchial carcinoma (4), hepatocellular carcinoma (2), Hodgkin lymphoma (1), pleural mesothelioma (1), urothelial carcinoma (2), sarcoma (1), renal cell carcinoma (1), and head and neck carcinoma (1). Twenty-one patients received anti-PD-1 monotherapy and ten patients were treated with anti-CTLA-4 and anti-PD-1 combination or sequential therapy. We divided the cohort up into two groups: patients who were diagnosed with N-irAEs (*n* = 20) and those who had other reasons for neurological deficits (*n* = 11).

Patients with N-irAEs (*n* = 20) suffered from different autoimmune neurological syndromes. In 9 patients, acute autoimmune polyneuropathy mimicking Guillain-Barré syndrome (GBS) was described. In six patients, N-irAEs were characterized as myositis and/or myasthenic syndrome. Another five patients were diagnosed with encephalitis or cerebellitis. Differential diagnoses (DD group) consisted of spinal or cerebral metastases (*n* = 7) and cerebrovascular disorders (*n* = 4).

The groups did not differ in terms of age or latency of symptom onset (Table 1). The interval between first administration of ICI therapy and final diagnosis of neurological symptoms varied from two weeks to 15 months within the N-irAEs group and from four weeks to 22 months within the differential diagnosis group (Table 1). Both groups exhibited a very similar gender distribution with a proportion of 65% male patients in the N-irAEs group and 64% in the differential diagnoses group. Regarding the systemic inflammation parameters, 50% of the N-irAEs patients presented with elevated CRP and 30% of them exhibited elevated white blood cell count. Only 2 patients of the N-irAEs group (10%) had fever. In the differential diagnosis group, 18% of patients showed an increased CRP value and an increased white blood cell count. None of the patients presented with fever at the onset of neurological symptoms.

#### 3.1.1. Case 1

**Figure 1:**
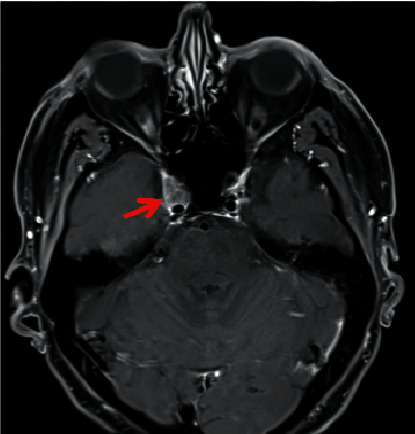
Brain MRI of Case 1. The red arrow displays metastasis within the right cavernous sinus.

A 63-year-old man was diagnosed with pulmonary metastasized hypopharyngeal carcinoma in 2018. Radiochemotherapy was not tolerated and thus treatment with PD-1 inhibitor nivolumab was started. After the fourth cycle, he reported double vision and a dropping right eyelid. Brain MRI was normal, and thus, his oncologist suspected an irAE. Prednisolone (100 mg/day) did not improve the patient's symptoms. Therapy with intravenous immunoglobulins was performed but symptoms further worsened. The patient was transferred to the neurological department. Neurological examination showed an extensive abduction deficit of the right eye, right-sided ptosis, and anisocoria (right > left). CSF analysis revealed slightly disturbed blood-CSF-barrier function (Qalbumin 8.6, protein 409 mg/l); while cell count (2/*μ*l) and lactate (2.1 mmol/l) were normal. OCB were negative. Serum Anti-Jo-1-antibodies indicating autoimmune myositis were found. However, despite immunomodulatory therapy, symptoms further deteriorated. Repeat high-resolution brain MRI was performed (Figure 1) and revealed metastasis formation within the right cavernous sinus reaching the foramen ovale as the cause of the patient's deficits. The patient was then referred for palliative radiotherapy.

#### 3.1.2. Case 2

**Figure 2:**
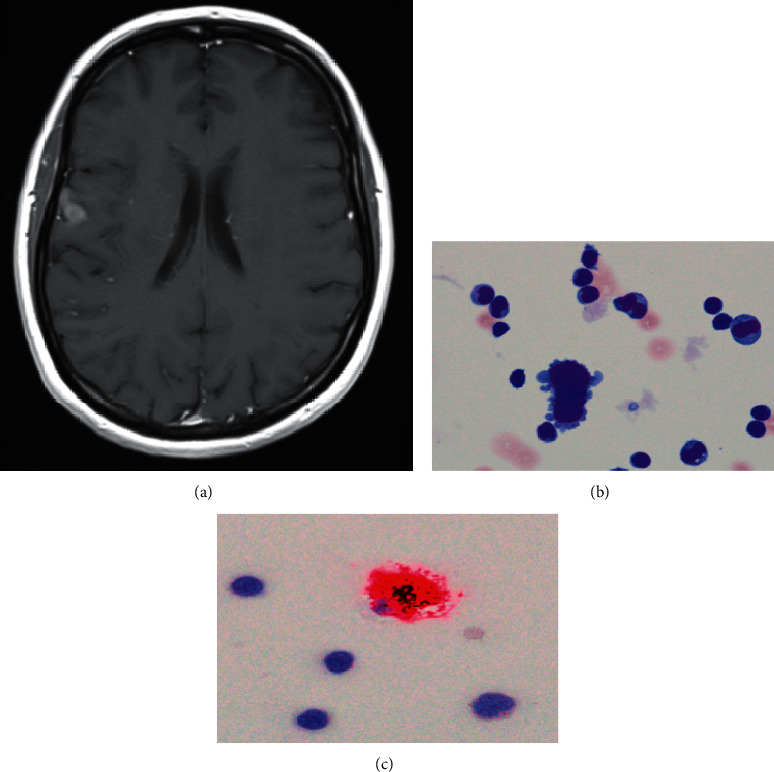
Diagnostic results of case 2. (a) Brain MRI with contrast enhancing lesion parietal right. (b) CSF cytology with melanoma cells, Pappenheim stain. (c) Proof of melanoma cells within CSF via melanin staining.

A 55-year-old female patient with metastatic malignant melanoma presented to the neurological department with sensory disturbance in the right hand and both legs. She reported that the symptoms had already started 3 months prior to first neurological examination. The patient had been treated with ipilimumab and nivolumab for five months. Fifteen months before neurological symptoms occurred, treatment was changed to targeted therapy with dabrafenib and trametinib because of progressive disease with pulmonary and cerebral metastases. Brain MRI one month prior to the first neurological presentation showed two cerebral metastases in the right parietal and frontal lobe. Further diagnostic steps were initiated as paresis of the right leg and coordination deficits of the right hand became obvious and could not be explained by the MRI findings. In addition to the known cerebral metastases (Figure 2(a)), actual brain MRI demonstrated further malignancy in the left pre-central gyrus. CSF analysis showed normal cell count (2/*μ*l), a slightly disturbed blood-CSF-barrier function (Qalbumin 6.9, protein 545 mg/l), and positive OCB (type 2). CSF cytology revealed malignant cells which were positive for the marker melanin (Figures 2(b) and 2(c)) indicating meningeosis neoplastica. Cerebral metastases were treated via stereotactic radiation and targeted therapy was changed to another therapeutic trial with ICI.

### 3.2. MRI and Electrophysiological Measurements

In patients without autoimmune adverse events (DD group), MRI detected brain and spinal column metastases in five patients and cerebrovascular events in four other patients (Table 2). Patients with metastases in the cavernous sinus (*n* = 1, see case 1) or in the brain stem (*n* = 2) showed distinct neurological deficits such as ptosis, oculomotor dysfunction, or facial nerve palsy initially suggesting a myositis-myasthenia-like syndrome. The two patients with spinal column metastases presented with pain, paraparesis, or bladder dysfunction. Intracerebral bleeding was found in two patients, while ischemic stroke was detected in another two patients. The two patients with intracerebral bleeding presented with vertigo, dysarthria, and coordination disorder of the left arm (*n* = 1) or with ataxia and right hemiparesis (*n* = 1), respectively. The ischemic strokes manifested as visual hallucinations and ataxia (*n* = 1), and as unspecific vertigo (*n* = 1). In patients with N-irAEs, MRI detected cerebral (*n* = 2) or spinal/osseous (*n* = 1) metastases. However, the metastases were considered rather an incidental finding, as they did not reflect the presenting symptoms suggesting a myasthenic and/or myositic syndrome. In three patients with demyelinating neuropathy, spinal MRI showed contrast enhancement of spinal nerve roots.

Electrophysiological findings were abnormal in 13 of the 16 N-irAEs patients examined (81%). Particularly, demyelinating changes were present in all six patients who suffered from acute neuropathy. Interestingly, such changes could also be detected in two subjects with myositic symptoms.

### 3.3. CSF Characteristics

**Figure 3:**
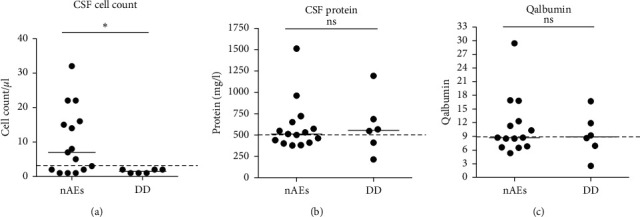
CSF characteristics of patients with N-irAEs compared to patients with other causes for their neurological complaints. (a) Comparison of CSF cell count/*μ*l. (b) Comparison of CSF protein (mg/l). (c) Comparison of Qalbumin. CSF: cerebrospinal fluid; DD: differential diagnoses; N-irAEs: neurological adverse events. Level of significance: ^*∗*^*p* < 0.05.

In 15 of 20 patients with N-irAEs, CSF analysis was performed, whereby Qalbumin was analyzed in 14/20. CSF cell count ranged from 1 to 32 cells/*μ*l (median: 7 cells/*μ*l), whereby nine patients (60%) showed an elevated cell count, defined as > 4 cells/*μ*l (Figure 3(a)). 6/11 patients within the DD group underwent lumbar puncture. None of the patients in this group, not even the patient with diagnosed meningeosis neoplastica, presented with an elevated cell count (range: 1–2 cells/*μ*l; median: 2 cells/*μ*l) (Figure 3(a)).

The amount of CSF protein did not differ between both groups. The mean CSF protein of the N-irAEs group amounted to 590 mg/l (range 376–1512 mg/l). The differential diagnoses group presented with a mean CSF protein of 497 mg/l (range: 213–1192 mg/l) (Figure 3(b)). In 8/15 patients (53%) in the N-irAEs group and in 4/6 patients (67%) in the DD group, an elevated CSF protein (defined as >500 mg/l) was detected. Elevated Qalbumin as an indication of blood-CSF barrier dysfunction was found in 10/14 (71%) and 4/6 (67%) patients, respectively (Figure 3(c)). 4/15 patients suffering from N-irAEs showed oligoclonal bands in CSF, three with type 3 and one with type 2, indicating intrathecal synthesis of immunoglobulin G. These patients presented with encephalitis (*n* = 2) or immune mediated neuropathy (*n* = 2). Within the group of other diagnoses, only one patient with oligoclonal bands was found. This patient was diagnosed with meningeosis neoplastica.

### 3.4. Autoimmune Antibody Patterns

In five patients with N-irAEs, either antineuronal- (*n* = 1), autoimmune encephalitis- (*n* = 1), or anti-acetylcholine-receptor antibodies (*n* = 4) were detectable (Table 3). Concentration of anti-acetylcholine-receptor antibodies ranged from 0.36 nmol/l to 11.9 nmol/l. One patient exhibited both anti-acetylcholine-receptor- and anti-Titin antibodies. One other patient showed both antineuronal antibodies, namely, anti-Sox-1 (titer 1 : 800) and autoimmune encephalitis antibodies (CASPR-2; titer 1 : 50), within one analysis. Interestingly, not all patients with myasthenic antibodies showed myasthenia gravis-like symptoms. In six cases, additional antibodies such as antinuclear antibodies (ANA), anti-SSA(Ro)-antibodies, or anti-GM-2 antibodies were found. All patients with detectable autoimmune antibodies suffered from N-irAEs (either myasthenic syndrome, myositis, or neuropathy). The only patient with measurable autoantibodies (namely, Jo-1-antibody) within the DD group was the one described as case 1 who turned out to be suffering from cavernous sinus metastasis.

### 3.5. Clinical Treatment and Outcomes of N-irAEs Patients

With three exceptions, all N-irAEs patients received immunomodulatory treatment. Eleven patients (55%) were treated with intravenous or oral steroid monotherapy, while four patients (20%) received a combination therapy of steroids and intravenous immunoglobulins. One patient each was treated with intravenous immunoglobulins or a triple combination of steroids, plasmapheresis therapy, and intravenous immunoglobulins. Under immunomodulatory therapy, 14/17 patients (82%) showed an improvement or complete remission of neurological symptoms. In four patients, the symptoms remained stable, whereas two of the four patients did not receive immunotherapy. One patient experienced a worsening of symptoms during immunotherapy and two patients died as a result of autoimmune neurological side effects. Both deceased patients clinically showed a myasthenic-myositis overlap syndrome with cardiac involvement.

## 4. Discussion

As ICI therapy rapidly evolves and indications are increasing, the numbers of reported ICI-induced autoimmune events are likely to increase as well. However, neurological symptoms occurring during or after ICI treatment still seem to be underreported [26]. A possible explanation could be that neurological deficits are rather unspecific such as muscle pain, ataxia, paresis, gait disorder, or impairment of the oculomotor system [18]. On the other hand, direct cancer associated symptoms have to be urgently considered and the diagnosis of N-irAEs is rather a diagnosis of exclusion in patients with ICI therapy [27]. Paresis of cranial nerves developing during ICI treatment may be especially difficult to distinguish from disease progression in routine clinical practice. As shown in case 1, symptoms caused by cavernous sinus metastases can mimic oculomotor symptoms that typically occur in ICI-associated myositis-myasthenia overlap syndromes [21, 28]. A misinterpretation may lead to unnecessary immunosuppressive therapy with steroids or even intravenous immunoglobulins. Therefore, dedicated high-quality brain MRI should be performed. In case of a normal MRI, the diagnostic process can be completed by CSF analysis and antibody measurements. Detailed cytological CSF examination is of particular importance with regard to the diagnosis of leptomeningeal metastasis since in those cases brain MRI as well as CSF cell count can be normal [29]. Our results demonstrate that various autoantibodies can be found in patients with N-irAEs. However, not in all patients of this cohort were autoimmune antibodies and clinical symptoms congruent. This observation supports the suggestion that N-irAEs do not reflect a specific neurological syndrome but represent an overlap syndrome of different clinical disorders like myositis, myasthenia gravis, and autoimmune polyneuropathy [21]. In general, the detection of autoantibodies suggests the presence of N-irAEs and makes alternative diagnoses less likely. Especially in life-threatening cases, the reliable distinction between N-irAEs and differential diagnoses is crucial. Of particular note, all patients in our clinic who died from N-irAEs, or showed a worsening of symptoms, also exhibited cardiac involvement with elevation of cardiac enzymes. Cardiotoxicity often appears in the form of ICI-related myocarditis [30] and is the most lethal manifestation of irAEs, irrespective of pneumonitis [31]. As ICI-related myocarditis is commonly associated with concomitant N-irAEs like myositis or myasthenic syndrome [32], neurologists should consider this a severe complication. Cardiac troponin appears to be a valid marker for manifest myocarditis with a sensitivity of 94–100% [33] and should be checked at an early stage in all patients with ICI-induced neuromuscular adverse events.

In our cohort, 31 patients were initially suggested to suffer from immune mediated disorders but an ICI-induced AE toxicity was found in only 20 of these patients. 11 patients had other reasons for new neurological deficits such as spinal or cerebral metastases or cerebrovascular disorders. Four patients presented with either intracerebral bleeding or ischemic lesions. This may be due to the fact that our patients are at an age when cerebrovascular diseases are rather frequent. However, there is evidence for a protective role of PD-L1 in intracerebral hemorrhage [34] and it seems to play an essential role in the neuroprotection afforded by regulatory T-cells against cerebral ischemia by mediating their suppressive effect [35]. None of the patients with intracerebral bleeding was diagnosed with brain metastases or received radiotherapy of the brain. Further investigations are needed to determine whether checkpoint inhibitors can promote intracerebral hemorrhage and/or cerebral ischemia.

**Figure 4:**
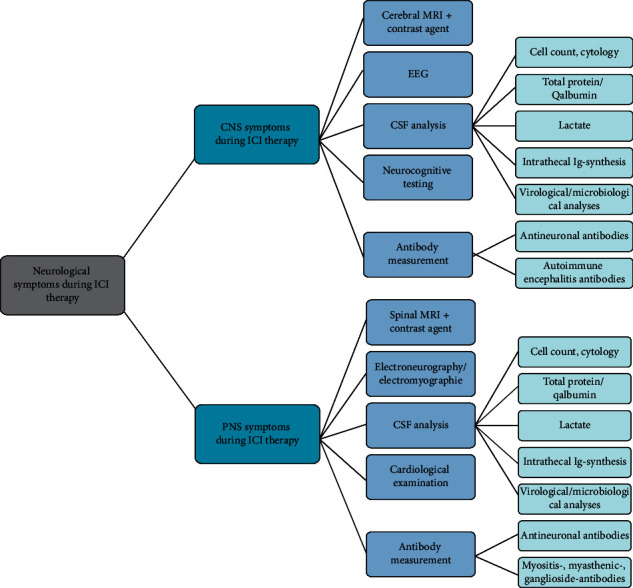
Diagnostic algorithm in case of neurological symptoms during ICI therapy. CNS: central nervous system; CSF: cerebrospinal fluid; EEG: electroencephalogram; ICI: immune checkpoint inhibitor; MRI: magnetic resonance imaging; PNS: peripheral nervous system.

Due to difficulties in clinical presentation, results of the CSF analyses are needed to better differentiate between the groups. In those cases where CSF analysis was conducted, CSF cell count was significantly higher in patients with ICI-induced N-irAEs compared with the differential diagnosis group. However, as 40% of our patients with N-irAEs displayed a normal CSF cell count, an inconspicuous CSF analysis does not exclude an autoimmune etiology of neurological symptoms during ICI therapy. In our cohort, CSF protein or Qalbumin as indicators of a disturbed blood-CSF-barrier function did not help to distinguish between autoimmune and non-autoimmune etiology of symptoms. An overview of a possible diagnostic algorithm in case of occurrence of neurological symptoms during ICI therapy is shown in Figure 4.

To date, there are still no uniform guidelines for the treatment of N-irAEs in patients with ICI therapy. We recommend, after exclusion of differential diagnoses, initial therapy with high-dose steroids for neurological symptoms with relevant patient impairment. In the absence of clinical improvement, complementary immunomodulatory therapies, e.g., with intravenous immunoglobulins, should be used. A group of particular relevance are those patients with indications of myasthenia-myositis-overlap syndrome. Due to the high risk of a fatal course, a combination therapy should be sought from the beginning. If the symptoms are pronounced, plasmapheresis therapy can also be considered initially.

Our study has several limitations. This work is based on a retrospective analysis of clinical data at a single institution. Due to the nature of this investigation, no causal relations can be made. Moreover, N-irAEs are primarily a diagnosis of exclusion. For most of our patients, N-irAE is the most obvious explanation for the neurological symptoms, but we lack unequivocal proof of the accuracy of the diagnosis. As a result, these findings need to be verified in larger cohorts, e.g., within a multicenter registry.

## 5. Conclusions

Autoimmune neurological events are rare but might be severe and even fatal complications of ICI therapy. Because symptoms are often unspecific, they can mimic other tumor-induced diagnoses such as metastases. Detailed investigations in neurological departments, in cooperation with treating oncologists, are recommended in patients with new neurological symptoms, for appropriate evaluation and management.

## Figures and Tables

**Table 1:** Baseline characteristics of both groups (N-irAE and DD) in comparison.

	N-irAEs group (*n* = 20)	DD group (*n* = 11)	*p*-value
Mean age, years (range)	66 (23–89)	69 (51–82)	0.626
Sex (males), *n* (%)	13/20 (65)	7/11 (64)	0.943
Latency: ICI therapy first administration, first neurological symptoms (days, mean, and ranges)	137 (30–153)	235 (30–671)	0.233
Latency: ICI therapy last administration, first neurological symptoms (days, mean, and ranges)	13.1 (1–37)	25 (6–70)	0.115
Elevated CRP at manifestation of neurological symptoms, *n* (%)	10/20 (50)	2/11 (18)	0.100
Elevated WBC at manifestation of neurological symptoms, *n* (%)	6/20 (30)	2/11 (18)	0.563
Fever at manifestation of neurological symptoms, *n* (%)	2/20 (10)	0/11 (0)	0.163

Data are presented as mean value and range. DD: differential diagnoses; f: female; ICI: immune checkpoint inhibitor; m: male; n.c.: not calculated; N-irAEs: neurological adverse events; WBC: white blood cell count.

**Table 2:** Results of radiological and electrophysiological measurements.

		N-irAEs group	DD group
Brain MRI	Cerebral metastases	2/10 (20%)	5/10 (50%)
	Ischemia/ICB	0/10 (0%)	4/10 (40%)
Spinal MRI	Inflammatory changes	3/9 (33%)	0/2 (0%)
	Metastases	1/9 (11%)	2/2 (100%)
Ephys	Polyneuropathy	13/16 (81%)	3/6 (50%)

Numbers indicate positive patients/all patients examined. DD: differential diagnosis; ICB: intracerebral bleeding; Ephys: electrophysiological studies; MRI: magnetic resonance imaging; N-irAEs: neurological adverse events.

**Table 3:** Results of serum-antibody measurements.

	N-irAEs group	DD group
ANA	3/20 (15%)	0/11 (0%)
Antineuronal antibodies	1/13 (8%)	0/4 (0%)
Myositis antibodies	0/8 (0%)	0/2 (0%)
Myasthenic antibodies	4/10 (40%)	0/1 (0%)
Autoimmune encephalitis antibodies	1/11 (9%)	0/4 (0%)
Others	3/20 (15%)	1/11 (9%)

Numbers indicate positive patients/all patients tested. Ab: antibodies; ANA: antineuronal antibodies; DD: differential diagnosis; N-irAEs: neurological adverse events. ^*∗*^Others: GM-1-antibody (*n* = 1), Jo-1-antibody (*n* = 1), Ro-antibody (*n* = 2).

## Data Availability

The datasets used and/or analyzed during the current study are available from the corresponding author on reasonable request.

## Abbreviations

AChR: Antiacetylcholine receptor
CNS: Central nervous system
CSF: Cerebrospinal fluid
CTLA-4: Cytotoxic T-lymphocyte associated antigen 4
DD: Differential diagnosis
DWI: Diffusion-weighted images
FDA: Food and Drug Association
FLAIR: Fluid-attenuation inversion recovery
GBS: Guillain-Barré syndrome
ICI: Immune checkpoint inhibitor
irAEs: Immune-related adverse events
MRI: Magnetic resonance imaging
N-irAEs: Neurological adverse events
OCB: Oligoclonal bands
PD-1: Programmed cell death protein
SWI: Susceptibility-weighted images.
